# Measurement of soil water characteristic curve using HYPROP2

**DOI:** 10.1016/j.mex.2020.100840

**Published:** 2020-02-22

**Authors:** Md Sami Bin Shokrana, Ehsan Ghane

**Affiliations:** Department of Biosystems and Agricultural Engineering, Michigan State University, East Lansing, MI, U.S.A.

**Keywords:** HYPROP2, Soil water characteristic curve, Subsurface drainage

## Abstract

Soil water characteristic curve (SWCC) has an important application in drainage, irrigation, soil physical behavior, and modeling hydrology and nutrient transport. However, measurement of the SWCC is often very time consuming, inaccurate and requires a lot of effort. In order to determine an accurate SWCC, we used HYPROP2. This method article extensively describes the topics which were not covered well by the instrument's manual such as collecting soil samples, use of the HYPROP refill unit, degassing water prior to degassing the tensio shafts and other procedures. Advice is provided in terms of better handling of the equipment to receive all four phases of an optimal measuring curve. Following the step-by-step procedure mentioned in this article would provide a high-quality SWCC. Our measurements were performed on both clay loam and sandy loam soils to show differences in the SWCC. We found that the upper tensio shaft took longer to cavitate for sandy loam soil compared to the clay loam soil.

• This paper describes an efficient and accurate method to determine the SWCC using HYPROP2.
• This method showed quick and reliable measurements of SWCC for a clay loam and sandy loam soil.
• This method includes procedure for soil sample collection and laboratory analysis with HYPROP2.

## Graphical abstract

**Figure:**
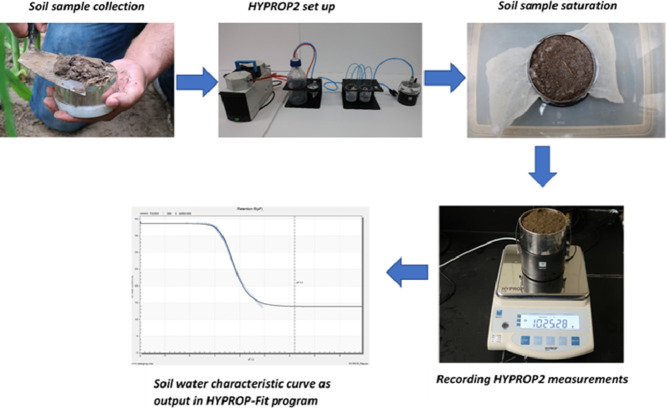

**Table:** Specification Table

Subject Area:	Agricultural and Biological Sciences
More specific subject area:	Soil and water
Method name:	HYPROP2 instrument
Name and reference of original method:	HYPROP manual (Meter Group)
Resource availability:	https://www.metergroup.com/environment/literature/?literature_category=3246

## Method description

This method explains the use of HYPROP (Hydraulic Property Analyzer) as an alternative technique to the conventional methods of measuring Soil water characteristic curve (SWCC) and unsaturated hydraulic conductivity of soil [8,18]. Measuring SWCCs have always been difficult due to the lack of sufficient data points using conventional methods and the amount of time these methods require to generate these curves. HYPROP2 follows an evaporation method where two tensiometers measure the tension implied by water to the soil column. Also, how the water content of the soil column changes over time at different tension values is observed [10,12,15]. However, the limitation of those tensiometers was that they would cavitate at a much smaller pressure typically between 70 and 90 kPa. A new design of tensiometers in the late 2000s allowed withstanding cavitation to a much higher tension values as high as 435 kPa [11,13,14,16,17]. HYPROP2 was developed based on those new designs of tensiometers.

HYPROP2 measures water potential at different soil saturation levels with the help of two tensiometers (i.e., one long and one short). This device is a more advanced version of HYPROP that has more robust and precise pressure transducers. It also introduces a faster optical monitoring of the measurements through a LED ring for better visualization of the current status of the device [8]. In HYPROP2, the soil sample stays on a laboratory balance during the experiment. Water evaporates from the soil over time, and HYPROP2 records the change of water potential during this process. This instrument also records the changing weight of the soil sample. This change of weight helps determine the moisture content of the soil. Finally, HYPROP-Fit program plots water potential against the changes in moisture contents to create a SWCC. The user should know that HYPROP2 can only measure the matric potential of the soil. It can measure water potential at the wet range of the soil, so the curve created with HYPROP2 will not be a complete curve. WP4C instrument can be combined with HYPROP2 to measure water potential in the dry range of the soil sample. WP4C is capable of measuring both matric and osmotic potential that completes the SWCC. However, HYPROP2 has its merits on producing high resolution data at the wet range of a soil sample.

There are studies where results obtained from HYPROP have also been compared with the results from conventional methods or to different models. Öztürk et al. [9] compared HYPROP outputs with the outputs from sand box (or pressure plate), which validated the use of HYPROP as a potential method for creating SWCC. Another study compared HYPROP with the BEST model (Beerkan Estimation of Soil Transfer Parameters) [6], which showed that the retention curves (SWCC) from HYPROP followed a faster and continuous dehydration process compared to the retention curves from the BEST model. Also, their comparison revealed that the results of soil water characteristic may vary based on the methodological approach used between different soil types. A different study evaluated the accuracy of HYPROP Measurement Systems (HMS) with HYDRUS-1D software package [1]. HYDRUS-1D can generate virtual pressure head in soil columns as a function of time, and independent tests were performed by this package on HYPROP system using the Van-Genuchten-Mualem model for a wide range of soil textures. The results from HYDRUS-1D showed that accurate estimates of SWCC and other parameters were obtained by HYPROP within the range of available retention measurements. Since HMS failed to operate at a very high pressure range, Wang et al. [20] evaluated the data points at a high-pressure range using centrifugal method. He added one control point at the high-pressure range of the SWCC already developed by HYPROP. The results showed that adding a control point to the high-pressure range makes the extrapolation of SWCC more reliable.

The step-by-step procedure to collect soil samples and to run HYPROP2 measurements is discussed in the later sections of this article.

### Soil sample collection

Soil samples were collected from two privately-owned farms. The first one is near Blissfield, Michigan. The soil type is predominantly Ziegenfuss clay loam, which is classified as a poorly drained soil [21] . The farmer uses a corn-soybean rotation in this field and applies commercial fertilizer. The second farm is near Palmyra, Michigan. The soil type is Brady and Macomb sandy loam at this farm, which is classified as a somewhat poorly drained soil [21]. The cropping system is corn-soybean rotation with commercial fertilizer application.

**Fig. 1:**
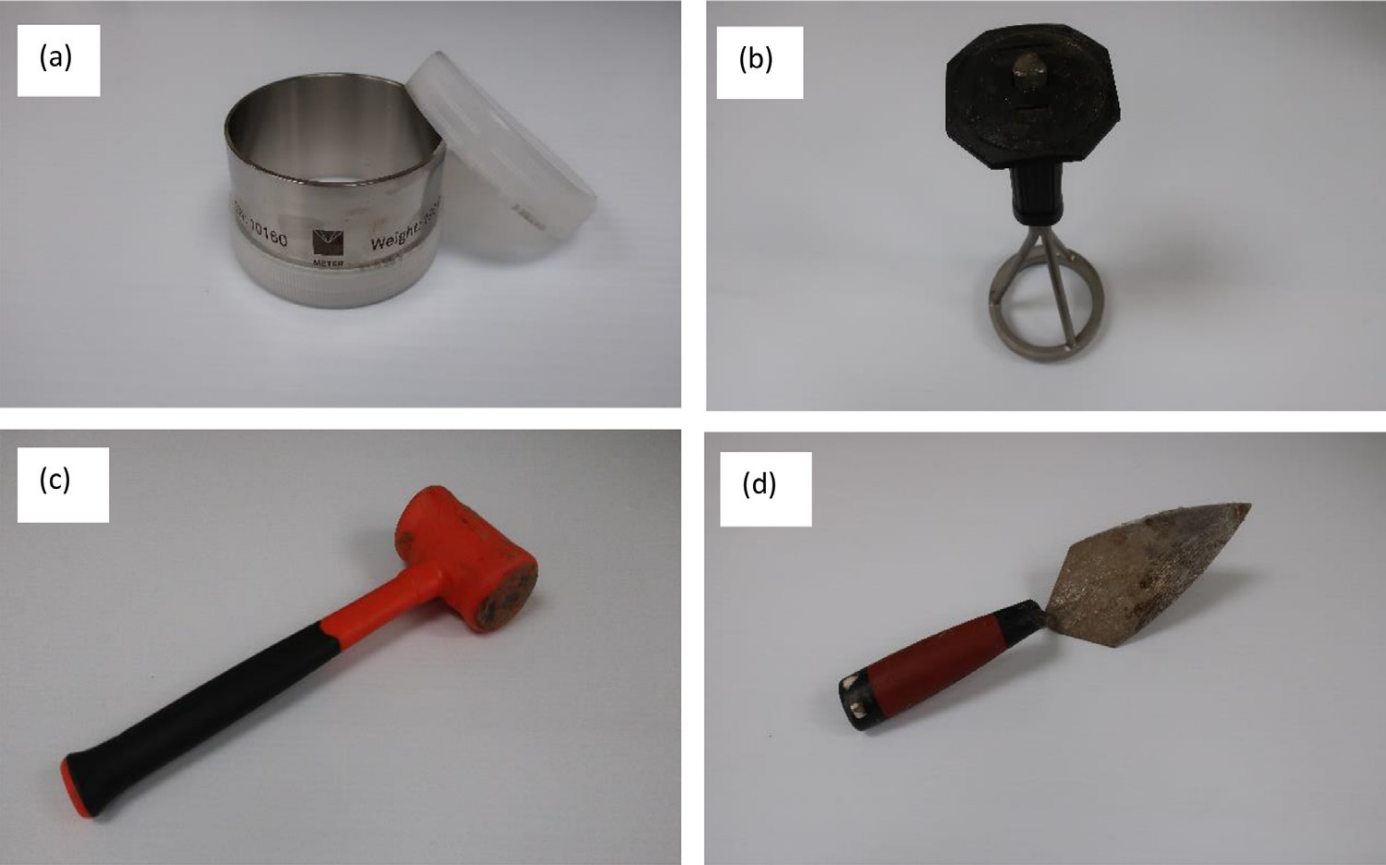
Soil sampling tools: (a) sampling ring, (b) sample ring insertion tool, (c) rubber mallet, and (d) trowel.

**Fig. 2:**
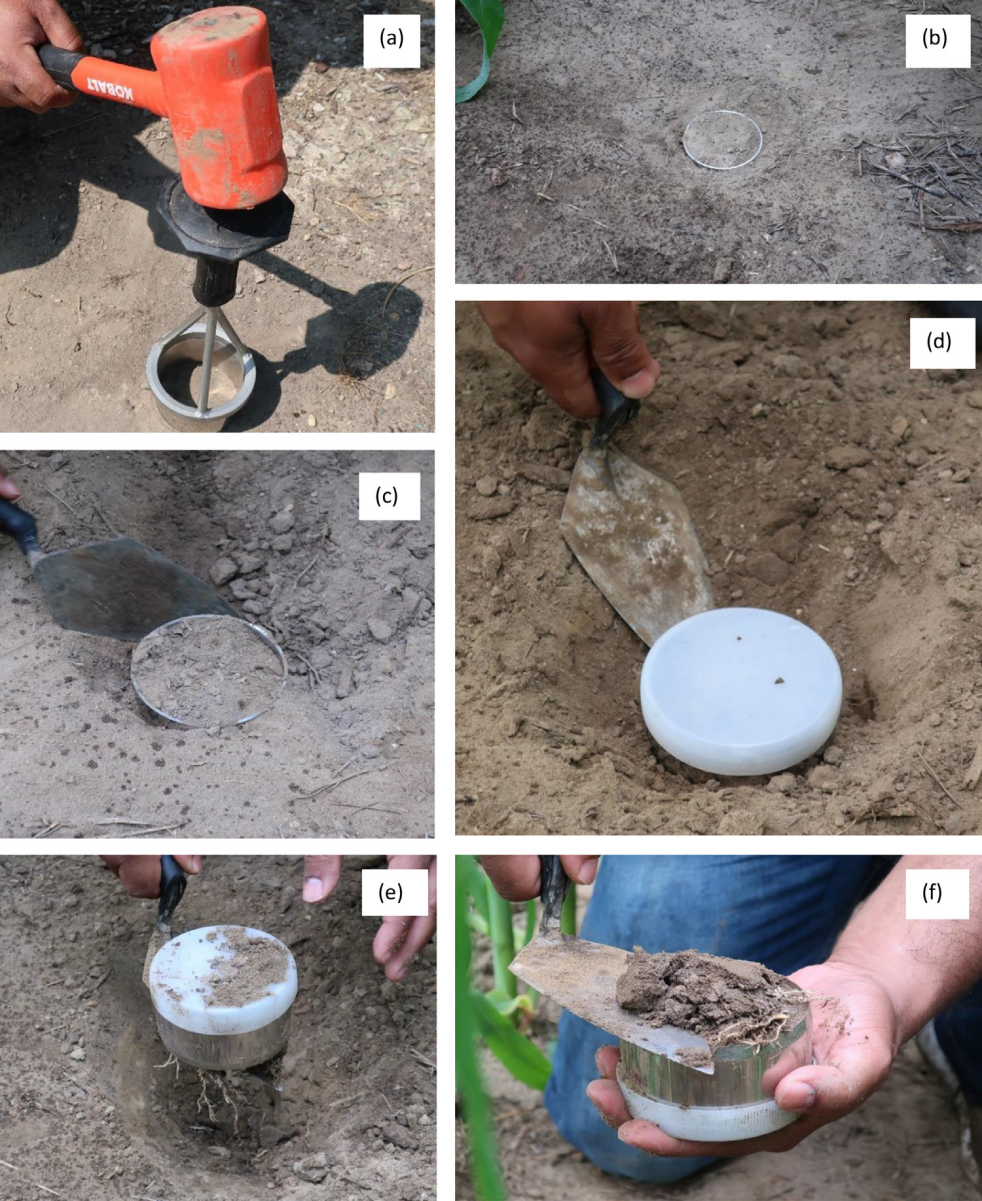
(a) hammering to collect soil cores, (b) soil core inserted and ready to collect after hammering, (c) removing soil from the sides of the sample ring to collect undisturbed samples, (d) put a white cap on the sample ring before digging it out with a trowel, (e) digging out the sample ring with trowel, and (f) flattening the soil surface with the trowel.

For both farms, soil samples were collected at two different locations with three replicates at each location. It is important to note that soil samples must remain undisturbed during collection. Four tools (Fig. 1) are needed to collect the soil samples: (i) a soil sampling ring (Meter Group), (ii) a sample ring insertion tool (Meter Group), (iii) a rubber mallet, and (iv) a trowel. Fig. 2 explains the whole soil sample collection process. Firstly, the soil sampling ring was attached to the sample ring insertion tool in such a way that the cutting edge of the ring was facing the soil surface. Then, the apparatus was hammered using the rubber mallet. The hammering was continued until the sampling ring had completely penetrated the soil. It is important to remember that the sampling ring just holds onto the insertion tool, and it cannot be attached to this without holding the bottom of the ring. Thus, whenever the ring had penetrated the soil, the insertion tool came off leaving the sampling ring inside the soil. Subsequently, a trowel was used to dig around the ring to loosen it up. Each ring comes with two white plastic caps to cover both ends of it. One of the caps were placed on the top surface of the ring. The trowel was put under the cutting edge of the ring, one hand was placed on the top of the ring, and the soil sampling ring with soil sample was taken out and was flipped. The excess soil was removed and leveled along the cutting edge using the trowel. Another cap was placed to cover the cutting edge of the ring. After collection, soil samples were placed in a box and transported to the lab for further analysis.

### Preparing HYPROP2

In this section, we will explain the steps required to prepare the HYPROP2 for determining the SWCC.

#### Degassing water

**Fig. 3:**
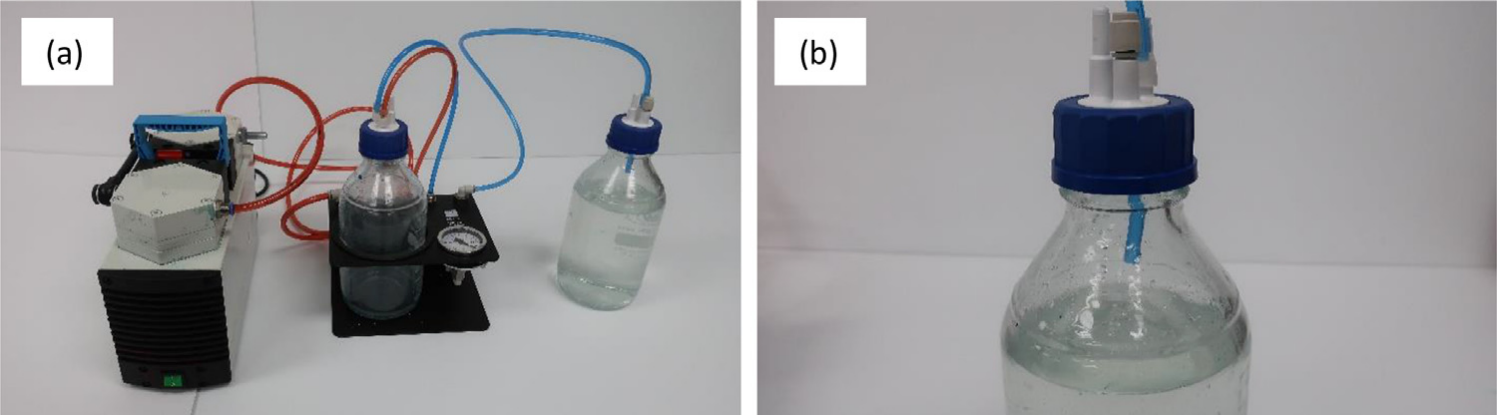
Degassing water using a vacuum pump and a vacuum bottle: (a) assembly of instruments for degassing DI water, and (b) blue tube should be kept in air inside the vacuum bottle while degassing DI water.

The first step in operating HYPROP2 is degassing water. A vacuum bottle was filled with deionized (DI) water. The tube coming out of the vacuum bottle was connected to the vacuum mount of the HYPROP2 system, and the vacuum mount was connected to the vacuum pump (Fig. 3(a)). Then, the pump was turned on to create vacuum in order to evacuate all the bubbles or gas from the DI water. It is important to remember that the tube inside the vacuum bottle needs to stay in the air (not submerged in DI water), so that it can evacuate as much air as possible from the bottle without removing water (Fig. 3(b)). The water was degassed for a couple of hours.

#### Degassing the HYPROP sensor unit

For running a HYPROP experiment, two of the devices need to be degassed completely: the HYPROP sensor unit and the two tensio shafts. There are two ways to perform degassing: (i) degassing the device using the HYPROP refill unit and (ii) degassing using syringes. Meter Group recommended degassing using the HYPROP refill unit because it is more accurate. Alternative to the HYPROP refill unit is manual degassing using syringes, which creates challenges to degas the water completely. This manual method requires a lot of labor, and chances of error is more than using the automated HYPROP refill unit.

The HYPROP manual gives a fair instruction about how to degas the device manually using syringes but does poorly on explaining the degassing process using HYPROP refill unit. The scope of this article is to give a better understanding of how the HYPROP refill unit works in degassing the tensio shafts and HYPROP sensor unit.

#### Degassing the sensor unit using the HYPROP refill unit

**Fig. 4:**
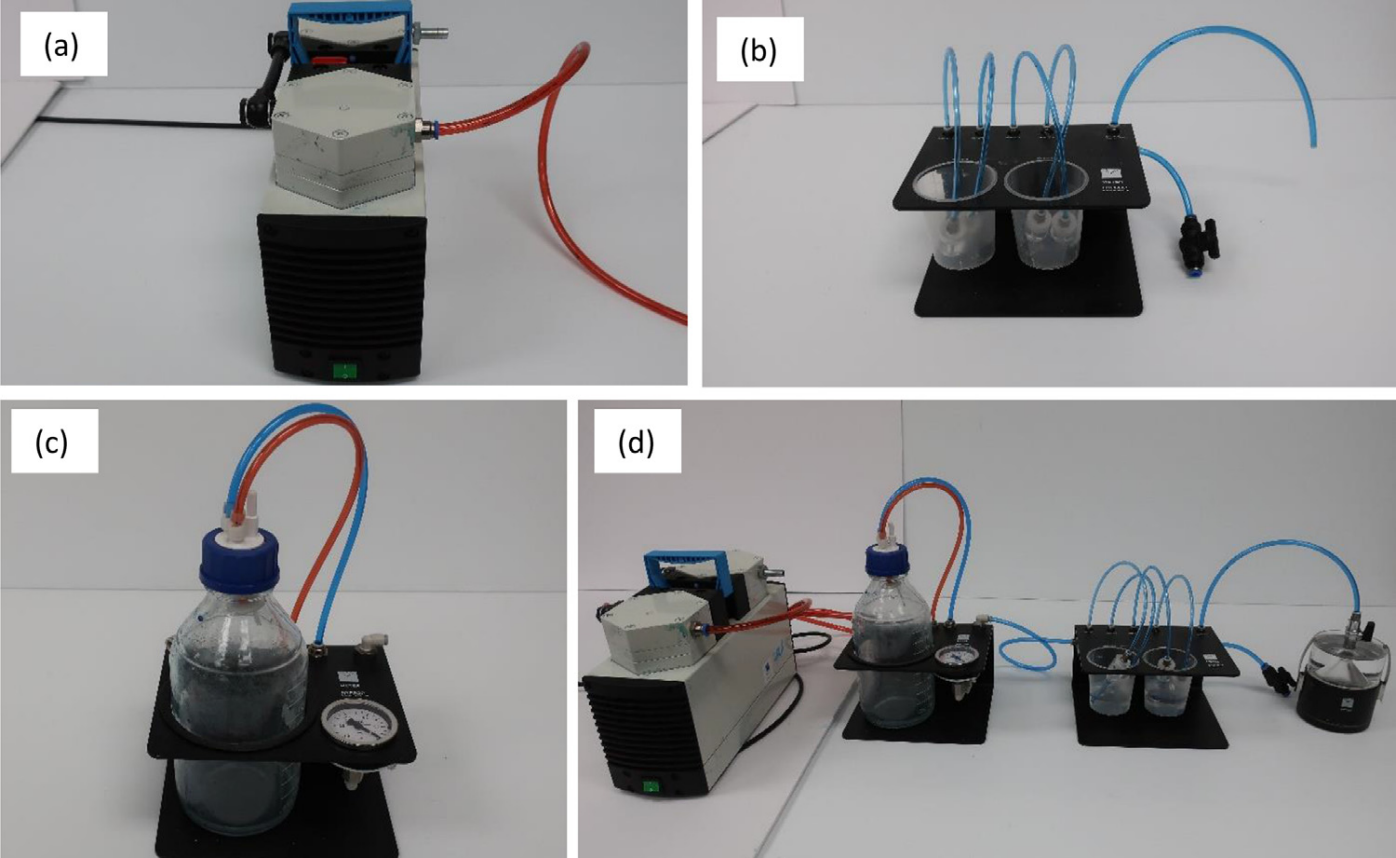
(a) vacuum pump, (b) beaker mount, (c) vacuum mount, and (d) assembly of Hyprop refilling unit.

*Assembly*: This method involves the use of a high-performance vacuum pump that can generate a vacuum pressure of around 0.85 to 0.90 bars, which can degas the sensor unit and both of the tensio shafts. The total arrangement consisted of four instruments connected to each other (Fig. 4). The first instrument was the vacuum pump, which was connected to a vacuum mount. The vacuum mount consisted of a pressure gauge and a vacuum flask. Whenever the pump was running, the vacuum pressure could be monitored looking at the pressure gauge. Also, when the degassing process took place, air bubbles from both tensio shafts and the sensor unit were collected in the vacuum flask to prevent water entering the pump. The vacuum mount was connected to a beaker mount where four tensio shafts can be degassed at the same time. The beaker mount has four ports. Each port is connected to a tube and each tube connects to an adapter. Finally, the beaker mount was connected to the HYPROP sensor unit. The top part of the HYPROP sensor unit was the acrylic adapter, which was attached to the HYPROP sensor unit base. The beaker mount was connected to the sensor unit with a tube.

*Procedure*: The degassing process using the HYPROP refill unit should be continued for about 12 to 24 hr. In our case, it was about 20 h. All the tubes were connected to their respective connections or ports (Fig. 4(d)). The color of the tubes should match the color of the connections, so that wrong tubes are not connected to wrong ports. Blind plugs were put in the connections not being used during the experiment. In the beaker mount, the beakers were filled with water from the vacuum bottle which had already been degassed. The tubes in the beaker mount were connected to the glass adapters and then each of the tensio shafts were screwed in the adapters. Then, the tensio shafts were placed in the beakers. Each beaker had a long and a short tensio shaft submerged in degassed water.

After placing the tensio shafts in the beakers, the black pressure valve attached to the beaker mount was closed by rotating it counterclockwise towards a vertical position of the knob. The pump was turned on for 10 min until the system reached full vacuum. Once full vacuum was achieved, the pump was turned off and the system retained the vacuum. The system then kept on degassing for about 2 h. After 2 h, the system started losing full vacuum, so the pump needed to be started again for 10 min to take the system back to full vacuum. If the user wants to perform this degassing process overnight, purchasing a programmable timer would be very helpful. This timer can run the pump for 10 min in every couple of hours and shut it off, thus saving time by running the degassing process overnight and performing the experiments during the daytime.

Getting full vacuum is extremely important for running experiments using HYPROP2. If a vacuum pressure value of around 0.85 to 0.90 bars (85–90 kPa) cannot be achieved, proper degassing will not be accomplished. Also, even if the system reaches full vacuum, the user needs to check if it can still hold the vacuum after the pump is turned off. The purpose of using the pump is to allow the system to reach full vacuum and then retain it for a couple of hours even if the pump is not in action. If the system is not able to hold the vacuum, there is definitely a leak in any of the refill unit components. A leak can happen in different ways such as a damaged vacuum bottle, the tubes may not be pushed all the way in through the ports of the vacuum mount and beaker mount, or the pressure valve attached to the beaker mount may be open. Thus, proper attention needs to be directed to these details.

#### Saturating the soil sample

After degassing the sensor unit using the HYPROP2 refill unit, the soil sample was saturated with water. The HYPROP manual suggests saturating the soil sample in degassed water. But in reality, the water for saturating soil sample solely depends on the purpose of the experiment. For agricultural applications, the soil core needs to be saturated in subsurface drainage water to simulate water movement in the soil matrix. Thus, we started saturating the soil cores in subsurface drainage water (collected from the on-farm sites). The required time of saturating different kinds of soils may vary (Table 1).

**Table 1:** Saturation time for different types of soils.

Soil Texture	Time for saturation
Clay loam	About 1 h^1^
Sandy loam	About 45 min^1^
Coarse sands	About 10 min^2^
Fine sands	About 45 min^2^
Silt	About 6 h^2^

1 Based on our experience.

2 Based on HYPROP manual.

**Fig. 5:**
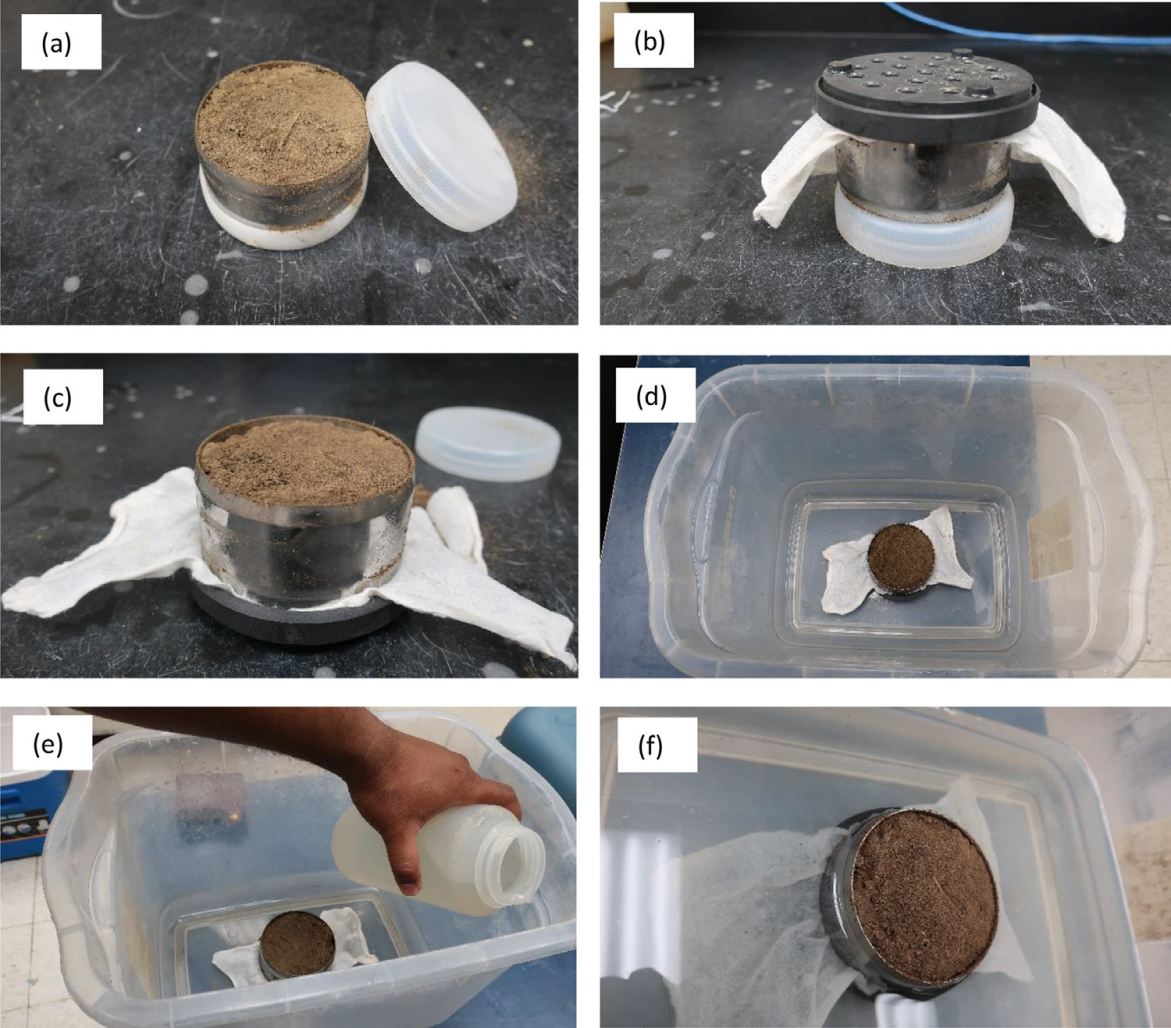
(a) Cap is removed from the cutting edge of soil core, (b) soil core is flipped, and a cheesecloth and perforated tray was put on the soil core, (c) soil core was flipped again with the cap removed, (d) soil core was put in a tray, (e) drainage water was poured in the tray, and (f) drainage water was poured until the water level reached the cutting edge of the tray.

A step-by-step saturation process of the soil sample is shown in (Fig. 5). The white cap from the blunt end of the soil core was removed. The soil core was covered with a cheesecloth, and the perforated tray was placed on top of the soil core. Then, the soil core was flipped, and the white cap was removed from the cutting-edge end of the soil core. Later, the soil core was placed in an empty tray. Drainage water was filled in the tray up to the cutting edge of the soil core. The user should be careful not to pour water on the top surface of the soil core to avoid trapping air. Also, pouring water from the top may immediately create a shiny surface indicating the soil sample is saturated although it may not be saturated in the middle. A white cap can be placed on top of the cutting edge to prevent evaporation and to protect the soil from solar radiation.

#### Preparing the programs

Once the soil was saturated, these two programs were used to determine the SWCC: (i) HYPROP-View and (ii) HYPROP-Fit. HYPROP-View was used during the beginning of the experiment to select the saving directories, selection of the HYPROP device, and to determine the data collection frequency from the sensor unit. HYPROP-Fit was used at a later stage to find the stop point and air entry point of the sample and further evaluation of the results. HYPROP-Fit allowed to select different computational methods for evaluation of results, and to export the results to the computer. Both programs were fairly easy to use, and we recommend using the HYPROP2 manual for more explanation.

#### Implementing tensio shafts in sensor unit

Once degassing was done, the valve connected to the beaker mount was opened very slowly. If the valve was opened too quickly, a sudden pressure shock could damage the pressure sensors in the sensor unit. Then, the tensio shafts were removed from the adapters. Once the tension shafts have been removed from the adapters, it is very important to keep the tensio shafts hydrated, so putting the silicone caps on them is a good practice. The user can also add degassed water on ceramic tips of the tensio shafts from time to time to prevent them from drying. The respective connection ports for each shaft is already drawn on the sensor unit.

**Fig. 6:**
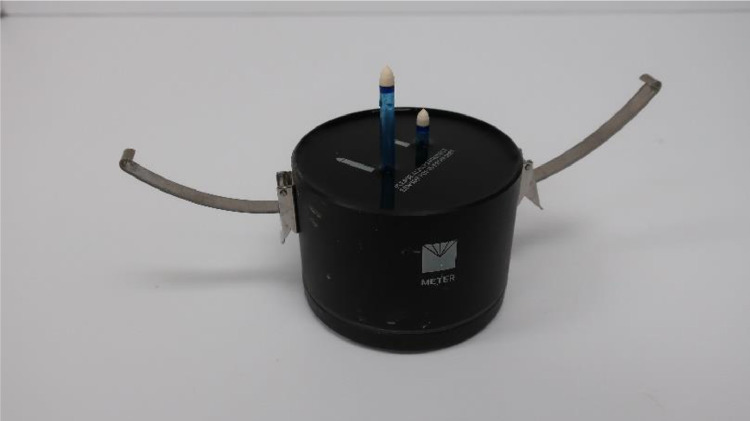
HYPROP2 sensor unit after implementing the tensio shafts.

When the tensio shafts are completely degassed and their ceramic tips are fully wet, they are ready to be screwed in the sensor unit. The HYPROP manual suggests that it takes about 9 turns for the tensio shafts to get sealed in the ports. From our experience, that was not always the case, or it was hard to measure exact 9 turns (Fig. 6). Thus, a better practice is to keep an eye on the HYPROP-View program where the refilling wizard shows how much pressure is being exerted by each tensio shaft. At the initial stages of screwing in the shaft, the pressure values remained small or negative, and the pressure started increasing with time. It is advisable to monitor the pressure values while screwing in the tensio shafts. Once it feels that the tensio shafts are close to sealing, they should be screwed in very slowly. Also, monitoring the pressure values in the HYPROP-View program will help avoid exceeding the maximum limit of 200 kPa (2000 hPa). The pressure values should always remain below this range. Once the shafts were fully screwed in the sensor unit, the silicone caps were removed. Then, a silicone gasket was put on the sensor unit to protect it from dust.

The tensio shafts are very sensitive and can get damaged by the slightest negligence while operating, so it is good to occasionally check whether the tensio shafts are working properly. An easy step to check whether the tensio shafts are functioning is to add small drops of water on the ceramic tip of the tensio shafts. The refilling wizard window in the HYPROP-View program will show the change in pressure due to addition of water droplets. If the pressure for both tensio shafts go back to zero in a little while, then the tensio shafts are functioning properly. This is to check the zero potential of the tensio shafts. Another way to check is to dry the ceramic tip with paper towel and to keep an eye to the pressure values. In this case, the pressure values should readily increase to the atmospheric air pressure.

*Precautions*: One important precaution before starting the experiment with HYPROP2 is to check whether offset recalibration is necessary. METER Group sets a narrow range of values for HYPROP sensors to function efficiently. If HYPROP has not been used for some time, the offset values of the electronic sensors may drift from the values set by METER Group. In these circumstances, the user needs to check for offset recalibration. From our experience, it was seen that during consecutive experiments the offset recalibration was unnecessary. However, the user should always check whether calibration is needed. Also, the HYPROP manual does a good job in explaining this calibration process. After degassing the sensor unit, the user needs to connect it with the provided USB adapter. Then, HYPROP-View program needs to be started. After clicking on the “refilling wizard”, the HYPROP sensor should be selected. Then, the software will check whether calibration is required. If calibration is needed, clicking the button “set zero value” will recalibrate the HYPROP sensors.

#### Assembling sensor unit to the soil sampling ring

**Fig. 7:**
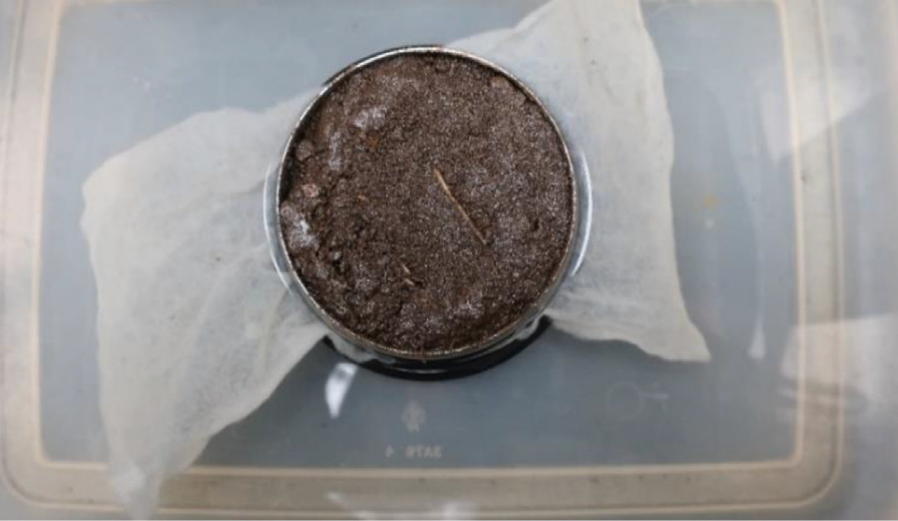
Shiny surface appears on top of the soil core.

When the tensiometers were fully functioning and the soil sample was saturated, i.e., a shiny surface appeared on top of the soil core (Fig. 7), it was time to attach the tensiometers to the soil core. The USB adapter was removed from the sensor unit, and the sensor unit was ready to be connected to the soil sampling ring. One important advice is to avoid removing the saturated soil sample from the tray filled with drainage water until the user is ready to start the measurements. For sandy soils, water drains through the soil core quickly, so the soil still might not be saturated while implementing the sensor unit to the soil core. A small auger and tensio shaft adapter were used for drilling holes in the soil core. The adapter should be set so that the user can remember which holes in the soil core are representing the long and short tensio shafts. HYPROP manual states that the adapter should be adjusted to the sample ring in such a way, so that the small hole of the adapter aligns with the sample ring number.

**Fig. 8:**
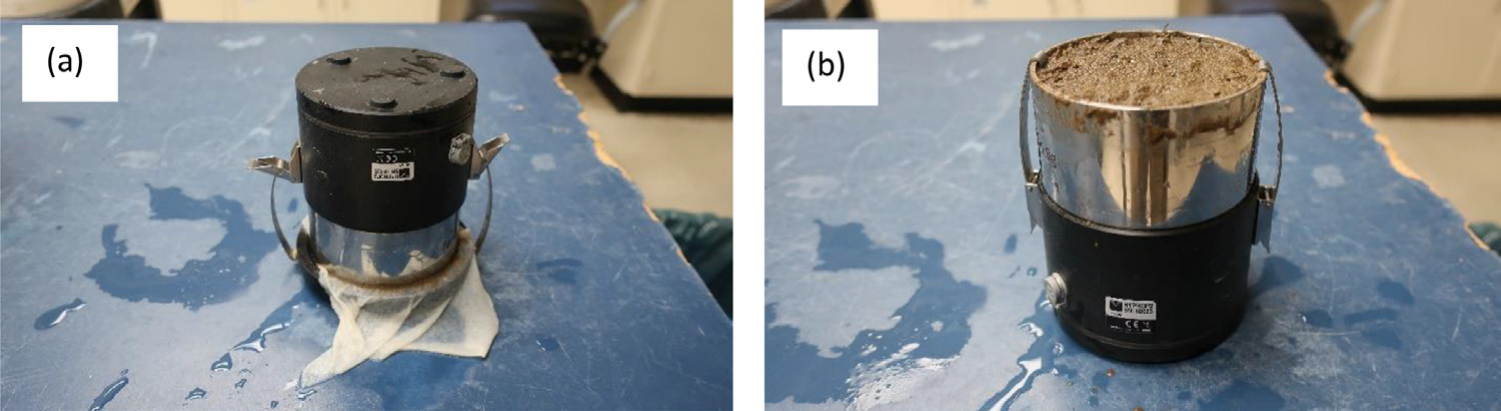
(a) sensor unit placed on the soil ring, and (b) after removing cheesecloth and perforated tray, the user flips the assembled sensor unit and soil core.

Once holes were made with the auger, there was a chance that air would enter those holes, so it was imperative to fill the holes again with water using the droplet syringe. At this stage, the soil was fully saturated and ready to be assembled to the sensor unit. Before assembling the soil core to the sensor unit, the silicone caps from the tensio shafts were removed. Then, the sensor unit was inverted and slowly placed close to the holes in the soil core. Once both tensio shafts were aligned with their respective holes in the soil core, the tensio shafts along with the sensor unit was slowly penetrated to the soil core (Fig. 8(a)). Next, the assembled sensor unit and sample ring was flipped (Fig. 8(b)) and the perforated bowl along with the cheesecloth was removed. Now, the ring is ready for the experiment.

### Starting the measurements

**Fig. 9:**
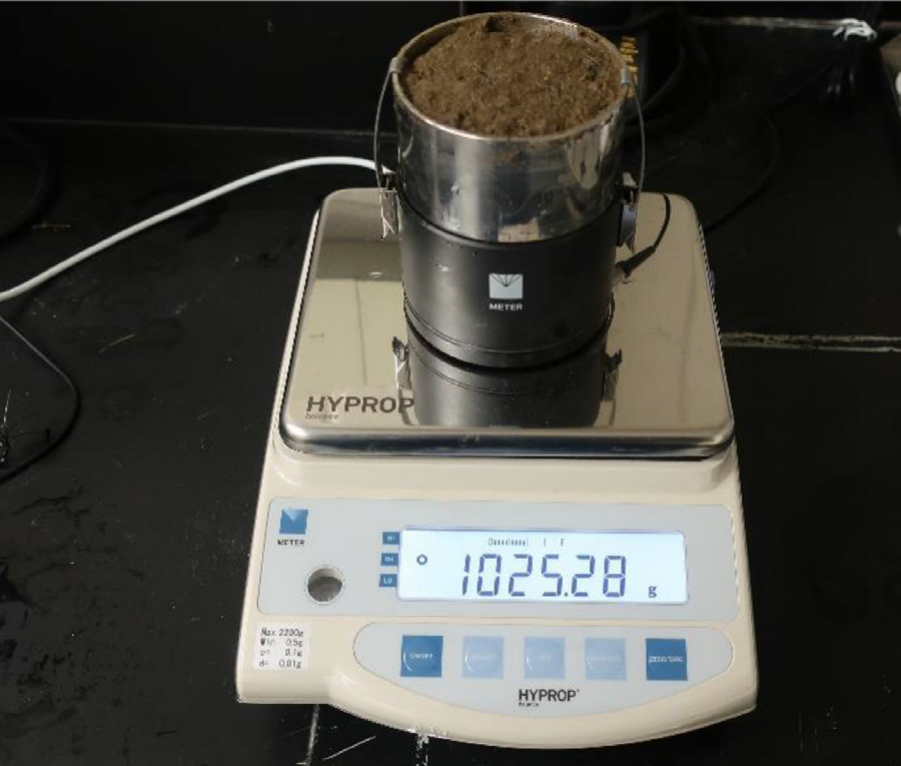
Assembled sensor unit and soil core is put on a balance to start measurements.

After the soil sampling ring was safely attached to the sensor unit, the combined piece was ready to be placed on a balance (Fig. 9). But before that, the balance was placed on a flat top and made level using the vertical screws and bubble. Next step was to calibrate the balance using the instructions in the HYPROP manual. The sensor unit was then placed on the balance. One end of a magnet clamp (i.e., comes with the HYPROP unit) was connected to the balance and another end to the sensor unit. A white USB cable (i.e., comes with the HYPROP unit) connected the balance to the computer.

Once both the sensor unit and the balance were connected to the computer, HYPROP-View program started recording change of weight of the soil core and pressure potentials at the long and short tensio shafts [8]. HYPROP-View allows selecting the frequency of the pressure potential data. The default value is 10 min, and it is possible to collect higher frequency data at different intervals during the experiment. The weight of the sampling ring (i.e., written on each ring) needs to be entered into the HYPROP-View program for correct evaluation of the results. These weight values are slightly different for each sampling ring. HYPROP-View also allows the user to select the units of pressure potential for example, Hectopascal (hPa) or Kilopascal (kPa) (1 kPa = 10 hPa). Usually, the experiment keeps on running for 2 to 7 days (depending on the type of soil) and data is collected continuously by the HYPROP-View program. The user can see the data in the HYPROP-Fit program without stopping the measurements. HYPROP-Fit also allows to look for the stopping point or air-entry point in the plot. Once this point is found, the measurements in the HYPROP-View program can be stopped.

### Stopping the measurements

**Fig. 10:**
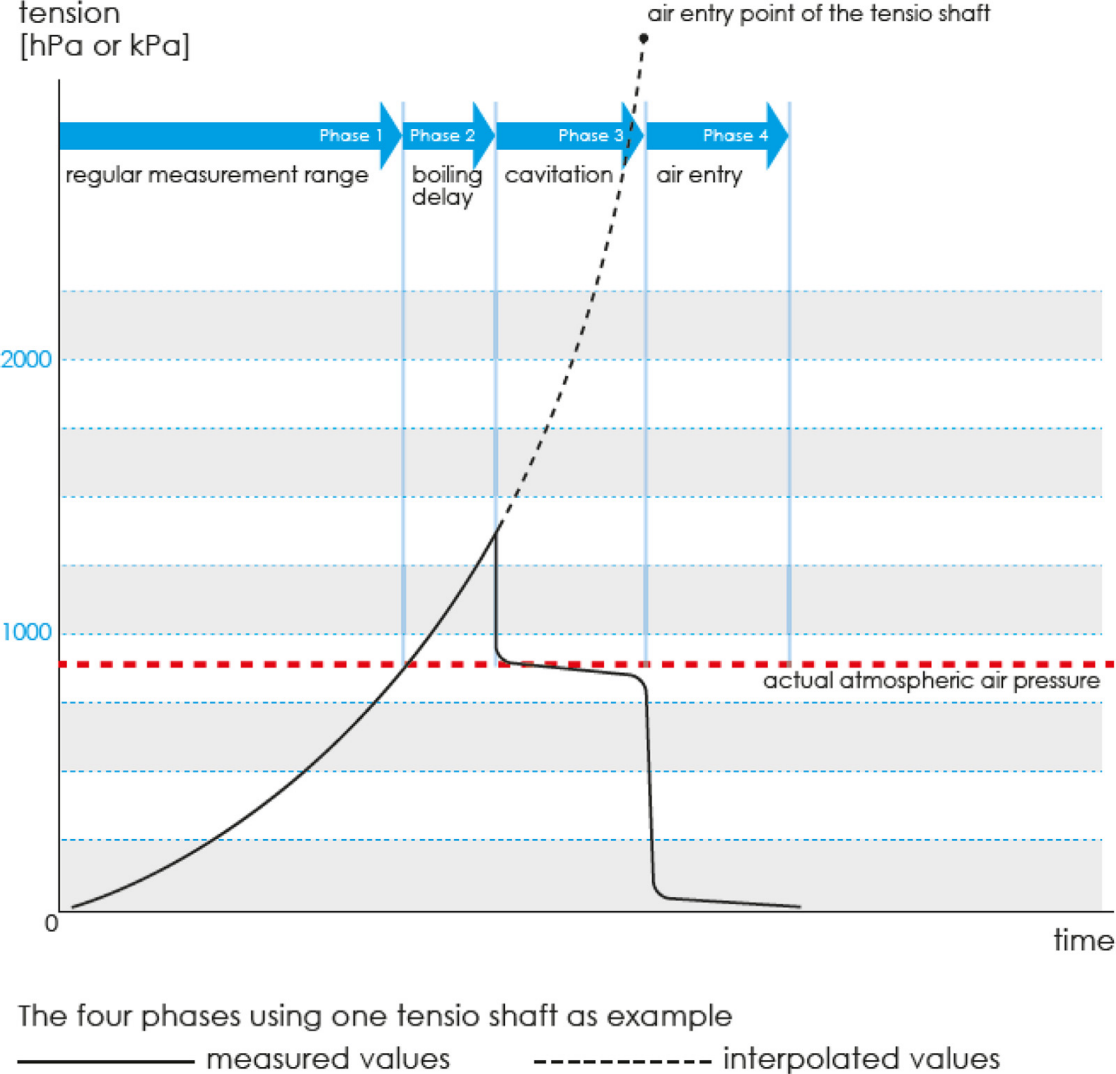
An optimal measuring curve for HYPROP measurements (image source: HYPROP Manual).

HYPROP measurements should be run for several days to create an optimal measuring curve with time. An ideal measuring curve (Fig. 10) is composed of 4 phases: (phase 1) regular measurement range, (phase 2) boiling delay phase, (phase 3) cavitation phase and (phase 4) air entry phase [8]. Phase 1 is the regular measurements where the tension values keep on increasing over time without showing any sign of decrease. In phase 2, tension is increased above the ambient air pressure, and the increase keeps on continuing until the tension value decreases. The boiling delay phase is often very difficult to achieve, but the results are still valid without this phase. Phase 2 can only be achieved if the user is able to completely degas the tensio shafts and the sensor unit, which would mean no air bubbles should be present in the system. In phase 3, some water vapor starts to form in the tensio shafts, and the tension values will have a sudden drop to the ambient air pressure, which is the point where the curve becomes flat. There will be very small decreases in the tension values after this phase. Air enters the ceramic tube at phase 4, and tension values instantly drop to zero.

After the tension values in the tensio shafts start dropping, a measurement can be manually stopped in three ways. First, using the stop point where the long tensio shaft reaches the cavitation phase, so the tension values do not increase anymore. The curve will become almost flat or reduce very little after this point. Second, using the air-entry point when the tension values at the long tensio shaft reduce to zero as air enters the ceramic tip. Third, using the air entry point when the tension values at both the long and short tensio shafts reach zero due to air entrapment in the ceramic tip.

To automatically stop the measurements, the user needs to click on the “search stop point” button in the HYPROP-Fit measurements tab. This program will detect a stop point in the tension curve of the top tensio shaft whenever it starts cavitating. It is important to remember that one should only use this “search stop point” option if the tensio shafts have been fully degassed and boiling delay phase have been achieved.

For our experiments, we were not able to achieve a boiling delay phase for both soil types due to presence of water bubbles in the tensio shafts even after degassing them for more than 20 h. When it is not possible to get a boiling delay phase, suboptimal curves develop, and the tension values will not go through a rapid drop. Instead, the tension values will become flat after increasing for some time. In this case, the automatic stop point detection using the HYPROP-Fit program may not be reliable. The user should manually find the stop point using the stop point cursor by moving right or left in the measurement tab of the HYPROP-Fit program. It is advisable to select the stop point at such a point where the slope of the tension curve is positive. In this paper, the same concept has been used to identify the stop point of tension curves for both clay and sandy loam soils.

### Determining the dry weight

After the experiment was ended, we determined the dry weight of the soil sample [8]. This dry weight helped to measure the volumetric water content based on how much weight was lost from the soil core due to evaporation. First, the soil core was detached from the HYPROP sensor unit, which required care because the tensio shafts were strongly attached to the soil core and a little extra force in the wrong direction could break them.

Once the soil core was detached from the sensor unit, it was placed in an aluminum tray. The weight of the empty tray was measured beforehand. It is normal that after several days of drying within ambient temperature, the soil becomes very crumbly (especially sandy soils), and the soil will easily fall off the sampling ring. To address this, the sampling ring was cleaned properly with a brush so that the tray could catch all the grains of the soil sample. The tray was placed in an oven for 24 h at 105C. After drying, the soil sample were weighed again. The dry soil weight was calculated by subtracting the empty aluminum tray weight from the combined weight of soil and tray after drying. This dry weight was recorded in the HYPROP-Fit program to measure the volumetric water content [7]. There are several soil hydraulic models available in the HYPROP-Fit program including Brooks-Corey, Fredlund-Xing, Kosugi, and van Genuchten models. The user can use any of these models, but the van Genuchten model is the most popular and the default model in the HYPROP-Fit program [2], [3], [4], [5].

## Method validation

**Fig. 11:**
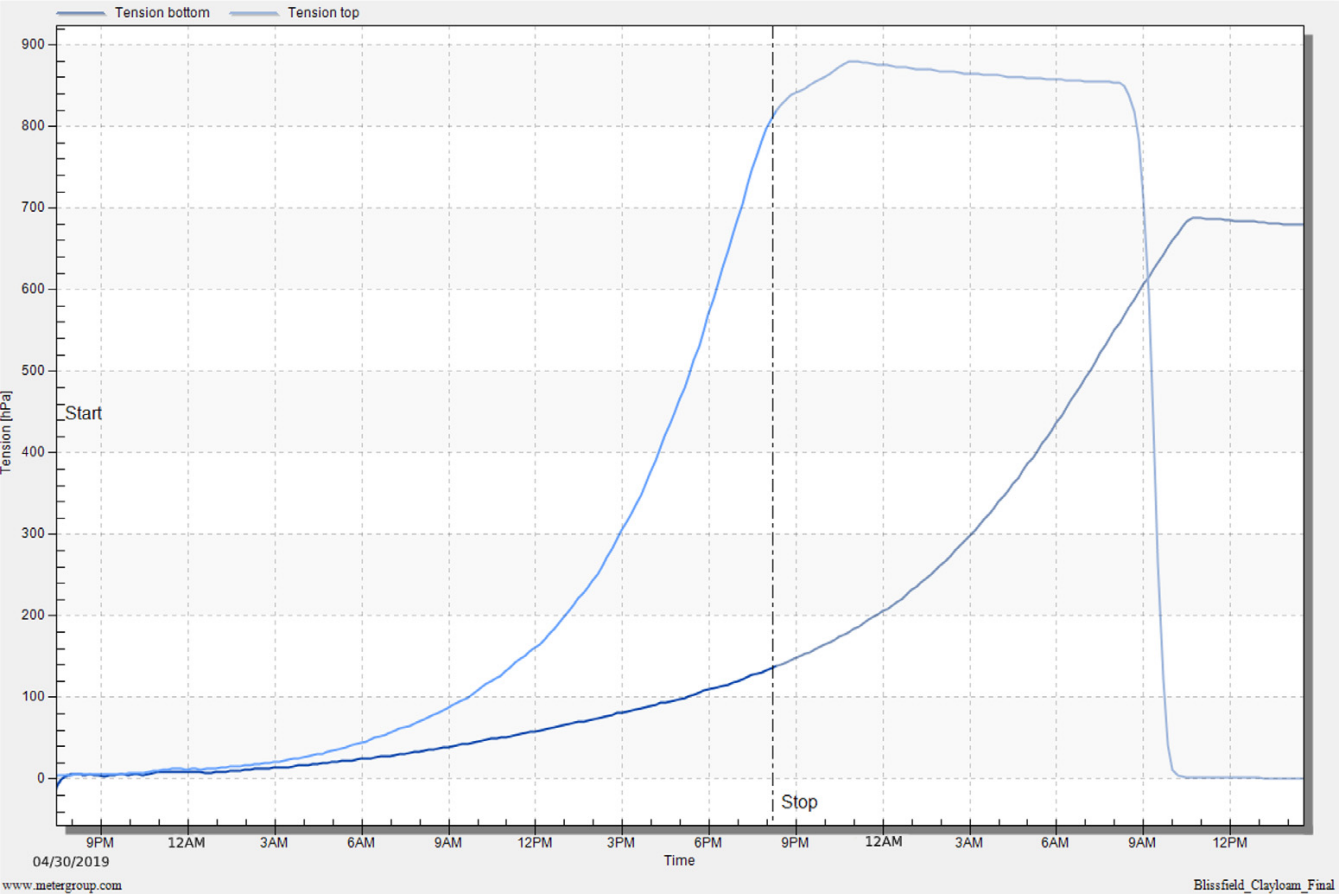
Tension curve for a clay loam soil. Tension is in units of hectopascal (1 kPa = 10 hPa).

**Fig. 12:**
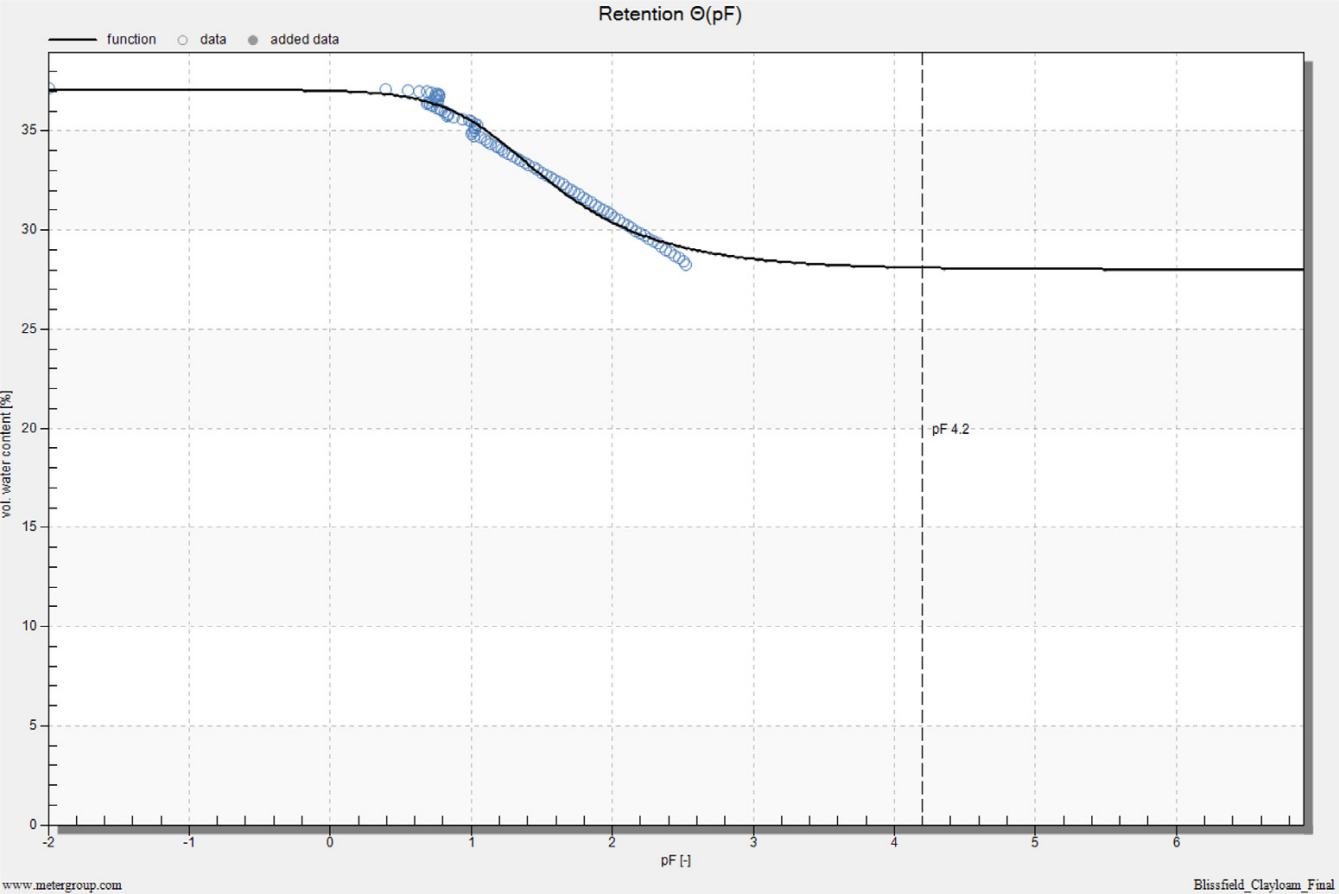
Volumetric water content vs. pF plot using evaporation method and fitted soil water characteristic curve for a clay loam soil.

**Fig. 13:**
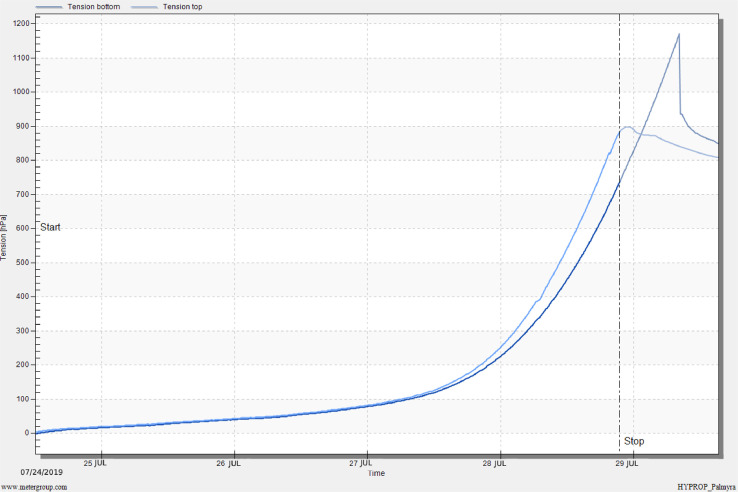
Tension curve for a sandy loam soil. Tension is in units of hectopascal (1 kPa = 10 hPa).

**Fig. 14:**
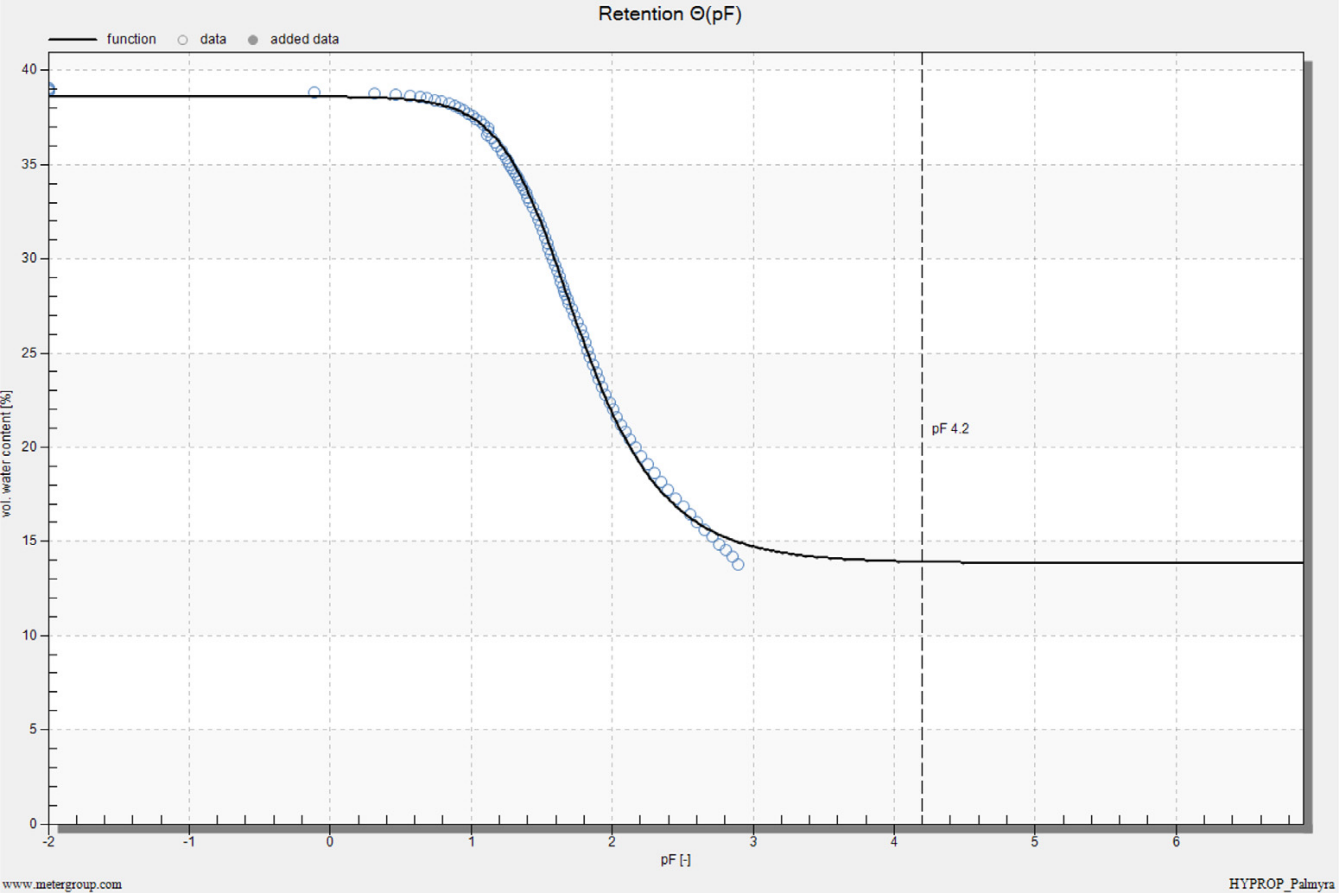
Volumetric water content vs. pF plot using evaporation method and fitted soil water characteristic curve for a sandy loam soil.

HYPROP measurements were conducted for both clay loam and sandy loam soils. The results for both soils are shown in Figs. 11 to 14 as part of our method validation. After finishing measurements, HYPROP2 produced one spreadsheet in Excel Worksheet (.xlsx) and five plots in image format (.png). The spreadsheet kept record of all the data measured during the experiment. The plots featured (i) change in tension values obtained from tensio shafts over time, (ii) retention or change in volumetric water content with change in pressure head (pF), (iii) change in unsaturated hydraulic conductivity with pressure head (pF), (iv) change in unsaturated hydraulic conductivity with volumetric water content, and (v) weight loss of undisturbed soil core over time.

### Clay loam soil

The water tension did not rise immediately at the beginning of the measurements, but rather both tensio shafts showed a very gradual increase and it was difficult to distinguish their pressure difference until about first 3 h of the measurements (Fig. 11). At 2 kPa (20 hPa), the tensio shafts were far enough away to determine the hydraulic conductivities. After 6 h of measurements, the tension values increased more rapidly than the beginning of the measurements and formed two gradually increasing curves. At this stage of gradual increment of curves, tension at the top shaft was higher than the tension at the bottom shaft. Both tension curves kept on increasing and after a day of measurements, the top tensio shaft reached its cavitation phase and air bubbles started entering the tensio shaft. This is the stage where the experiment should have been stopped, but measurements were not stopped, and the experiment was continued. Since the measurements were not stopped, the tension at the bottom shaft kept on rising and it started cavitating after 12 h. At this point, the soil sample had lost 9% of water over the drying process (Fig. 12). Since experiment was not stopped automatically using the “search stop point” option of the HYPROP-Fit program, the experiment was stopped by manually finding a stop point before the cavitation phase until where the slope of the tension curve of top tensio shaft kept on increasing without showing any sign of decrease (method 1 explained in *Stopping the measurements* section).

HYPROP-Fit program lets the user choose different models to analyze the HYPROP2 measurements. The default one is the Van Genuchten model (1980) where volumetric water content of the soil is calculated by the following equations

(1) $$S_{e}=\;\frac{\theta -\theta_{r}}{\theta_{s}-\theta_{r}}$$

(2) $$\text{and}S_{e}={\left[\frac{1}{1+{\left(\alpha h\right)}^{n}}\right]}^{m}$$

where *S_e_* is effective saturation (cm^3^/cm^3^), h is pressure head (cm), *ϴ* is the volumetric water content (cm^3^/cm^3^; multiply by 100 to get this value in percentage), *ϴ_r_* is the residual water content (cm^3^/cm^3^) and *ϴ_s_* is the saturated water content (cm^3^/cm^3^). Additionally, *α* is the shape parameter related to the inverse of the air entry pressure (cm^−1^), n is the shape parameter that controls both the bending of the retention curve at the air-entry region and the asymptotic curvature towards the residual water content, and *m* is the additional shape parameter which equals to 1 – (1/*n*).

It is also possible to select 4 other models (Brooks-Corey, Freudland-Xing, Kosugi and Van Genuchten mnvar) in HYPROP2 for analysis, but we used the Van Genuchten [4] model also known as the traditional constrained Van Genuchten–Mualem model for our study. After running this model by HYPROP-Fit program for a clay loam soil, values for the following parameters were generated as outputs:

*α* = 0.0730, *n* = 1.661, *m* = 1– (1/*n*) = 0.398, *θs* = 0.371 and *θr* = 0.280

Thus, the equation of volumetric water content for clay loam soil is calculated as

(3) $$\theta =0.091{\left[\frac{1}{1+{\left(0.0730*h\right)}^{1.661}}\right]}^{0.398}+0.280$$

HYPROP-Fit program also provides estimates of field capacity and permanent wilting point. Field capacity is estimated at 33 kPa matric potential. The water content at permanent wilting point is estimated at 1500 kPa. The above model provides the water content at 6 kPa (*h* = 63.1 cm, and pF=1.8), 33 kPa (*h* = 316.2 cm, and pF=2.5) and 1500 kPa (*h* = 15,848.9 kPa, and pF=4.2) to be 31.3%, 29.1% and 28.1%, respectively. These values are important for irrigation timing, and can be used as an input for hydrological models such as DRAINMOD [19].

### Sandy loam soil

At the beginning of the experiment, the water tension at both tensio shafts followed a gradual increase for the first 3 days (Fig. 13), and both the tensio shafts showed the same tension values during this time. At about 140 hPa (14 kPa), the tensio shafts were far enough away to determine the hydraulic conductivities. After about 4 days of measurements, the tension values increased at a greater slope. Top shaft showed higher tension value than the bottom shaft. The measurements were completed by the failure of upper tensio shaft after more than four days. At this point, the soil sample had lost about 25% of water over the drying process (Fig. 14). The experiment was stopped by manually finding a stop point on the top tension curve as explained in section 1.4.

After collecting the data for the sandy loam soil, HYPROP-Fit program gave parameter values as follows

*α* = 0.0302, *n* = 1.975, *m* = 1− (1/*n*) = 0.494, *θs* = 0.386 and *θr* = 0.138

Using the Van Genuchten-Mualem [4] model, the equation for this soil is calculated as:

(4) $$\theta =0.248{\left[\frac{1}{1+{\left(0.0302*h\right)}^{1.975}}\right]}^{0.494}+0.138$$

This model gives the water content at 6 kPa (*h* = 63.1 cm, and pF=1.8), 33 kPa (*h* = 316.2 cm, and pF=2.5) and 1500 kPa (*h* = 15,848.9 kPa, and pF=4.2) to be 31.3%, 29.1%% and 28.1%, respectively.

## Summary

This study provides an extensive guideline for using HYPROP2 including collecting an undisturbed soil sample and using the instrument to determine an accurate SWCC. The HYPROP2 manual does not give good instructions on how the soil samples should be collected and how to saturate those samples in water based on one's research needs. Also, this article talks about the degassing process using the HYPROP refill unit. Even though the optimal curve is expected from HYPROP2 based on the instrument manual, a suboptimal curve can also be used to determine the SWCC. This method is highly efficient in degassing water and to generate good results. Anyone correctly following this method article should be able to replicate the results obtained in this study.
